# Supplementary material for: A Potential Adhesin/Invasin STM0306 Participates in Host Cell Inflammation Induced by Salmonella enterica Serovar Typhimurium
Source: Int J Mol Sci. 2023 May 3;24(9):8170. doi: 10.3390/ijms24098170 (PMC10179656; doi:10.3390/ijms24098170)
Supplement: Supplementary file 1 [file ijms-24-08170-s001.zip › Supplementary Material.pdf]

Table S1. Primer sequences for molecular clone.

Primers	Primer sequences (5'→3')
0306-Kan-F	TCTGAAATGAAAAACTTTTTCGCAGTCTGCATCATTCCCCTTGTG GTAGCCTGGTCCGCTACTGCATCAGGCGATTGTGTAGGCTGGAG
0306-Kan-R	TTAAAAGGCGTAAGTAATGCCGAGCATGAAGTCATTGGAGGCAG CCTTTGTGTCTGCATCATAAGCGGTATAACGGCTGACATGGGAATTAG
0306-Y-F	GGCAGGTTCTGAAATGAAAAAC
0306-Y-R	CCGATAGTGTTTAAAAGGCGTAAG
0306-Hind III	CAGCTTATCATCGATAAGCTTTCTGAAATGAAAAACTTTTTCGCAGTCTGC
0306-EcoR V	GCTGTCGGAATGGACGATATCTTAAAAGGCGTAAGTAATGCCGAGC
0306-XhoI	TGGTGGTGGTGGTGGTGCTCGAGTCTGAAATGAAAAACTTTTTCGCAGTCTGC
0306-BamH I	CAAGGCCATGGCTGATATCGGATCCTTAAAAGGCGTAAGTAATGCCGAGC

Table S2. Primer sequences for qRT-PCR.

Species	Primers	Primer sequences (5'→3')
S. Typhimurium	0306-F	CCCTTGTGGTAGCCTGGTC
	0306-R	GCCTTTAGCATCCGTCTCA
	gyrA-F	CGGGATACAGTAGAGGGATAGCGG
	gyrA-R	CACCAACGACACGGGCAGATT
	csgA-F	ATGCCCGTAAATCTGAAACG
	csgA-R	CCGTATTGGCCGACAGTAAT
	csgD-F	TATGATGGAAGCGGATAAGAA
	csgD-R	GCACCCAGGCAGTTTCAT
	hilA-F	AAATATCCACGCAGGAAATAAC
	hilA-R	GGGCAACCAGCACTAACG
	hilC-F	ACCAAGCCCAGGCACT
	hilC-R	TGTGGTTATGATACGCCTTC
	hilC-F	TGTAAGTAATAGTCATCAGCGTCCTG
	hilC-R	CTCCGAAAGCAAACTAAAGTGAAA
	sopB-F	AGCGGGCGAGGCGGTAAG
	sopB-R	CCGGCTGGGTCAACGATTGC
	sopD-F	TAATGAAAGTCGGCTTGCTC
	sopD-R	TGCCCGTGAATGATGGAG
	sopE-F	ACTAACATAACACTATCCACCCAGCAC
	sopE-R	ACCGCCCTACCCTCAGAAGC
	sopE2-F	ACTAACATAACACTATCCACCCAGCAC
	sopE2-R	GCTTCTGAGGGTAGGGCGGT
	mipA-F	GTGGAATGACAATGCCGATAAGT
	mipA-R	GCCAGTGAGGTACGCAGGAA
Pig	TNF-α-F	GCATCGCCGTCTCCTACCA
	TNF-α-R	CCTGCCCAGATTCAGCAAAGT
	IL-1β-F	GAGCTGAAGGCTCTCCACCTC
	IL-1β-R	ATCGCTGTCATCTCCTTGCAC
	CXCL2-F	CGCTGCTGCTCCTGCTT
	CXCL2-R	GAGTGGCTATGACTTCCGTTT
	Occludin-F	ATCAACAAAGGCAACTCT
	Occludin-R	GCAGCAGCCATGTACTCT
	GAPDH-F	GTGAAGGTCGGAGTGAACGGATTT
	GAPDH-R	CCCATTTGATGTTGGCGGGAT
